# Molecular Characterization of a New Virus Species Identified in Yam (*Dioscorea* spp.) by High-Throughput Sequencing

**DOI:** 10.3390/plants8060167

**Published:** 2019-06-11

**Authors:** Gonçalo Silva, Moritz Bömer, Ajith I. Rathnayake, Steven O. Sewe, Paul Visendi, Joshua O. Oyekanmi, Marian D. Quain, Belinda Akomeah, P. Lava Kumar, Susan E. Seal

**Affiliations:** 1 Natural Resources Institute, University of Greenwich, Central Avenue, Chatham Maritime, Kent ME4 4TB, UK; M.Bomer@greenwich.ac.uk (M.B.); A.I.RathnayakeMudiyanselage@greenwich.ac.uk (A.I.R.); S.O.Sewe@greenwich.ac.uk (S.O.S.); paul.muhindira@qut.edu.au (P.V.); S.E.Seal@greenwich.ac.uk (S.E.S.); 2 International Institute of Tropical Agriculture (IITA), Oyo Road, PMB 5320, Ibadan, Nigeria; J.Oyekanmi@cgiar.org (J.O.O.); L.Kumar@cgiar.org (P.L.K.); 3 Council for Scientific and Industrial Research-Crops Research Institute (CSIR-CRI), Fumesua, P. O. BOX 3785, Kumasi, Ghana; marianquain@hotmail.com (M.D.Q.); belindaakomeah@gmail.com (B.A.)

**Keywords:** RNA-Seq, virus detection, *Betaflexiviridae*, next-generation sequencing, HTS

## Abstract

To date, several viruses of different genera have been reported to infect yam (*Dioscorea* spp.). The full diversity of viruses infecting yam, however, remains to be explored. High-throughput sequencing (HTS) methods are increasingly being used in the discovery of new plant viral genomes. In this study, we employed HTS on yam to determine whether any undiscovered viruses were present that would restrict the international distribution of yam germplasm. We discovered a new virus sequence present in 31 yam samples tested and have tentatively named this virus “yam virus Y” (YVY). Twenty-three of the samples in which YVY was detected showed mosaic and chlorotic leaf symptoms, but *Yam mosaic virus* was also detected in these samples. Complete genome sequences of two YVY viral isolates were assembled and found to contain five open reading frames (ORFs). ORF1 encodes a large replication-associated protein, ORF2, ORF3 and ORF4 constitute the putative triple gene block proteins, and ORF5 encodes a putative coat protein. Considering the species demarcation criteria of the family *Betaflexiviridae*, YVY should be considered as a novel virus species in the family *Betaflexiviridae*. Further work is needed to understand the association of this new virus with any symptoms and yield loss and its implication on virus-free seed yam production.

## 1. Introduction

Yam (*Dioscorea* spp.) is a preferred staple food for over 90 million people in West Africa, with this region contributing over 95% of the world’s total yam production [1]. Nigeria and Ghana are the major producer and exporter of yams worldwide, respectively. Yams are mainly produced by small-holder farmers who rely on the crop for food and income security. Farmers rely on obtaining their planting material either from their own farms, or by buying the surplus from neighbouring farmers. This means that the planting material is often of low quality and infected with several pathogens, mainly viruses.

Despite its importance and high value, yam productivity is compromised severely by the impact of yam viruses and the unavailability and associated high costs of high-quality clean seed yam [2]. To date, numerous different virus species belonging to the genera *Aureusvirus*, *Badnavirus*, *Carlavirus*, *Comovirus*, *Cucumovirus*, *Fabavirus*, *Macluravirus*, *Potexvirus* and *Potyvirus* [3,4,5,6,7] have been reported and characterized in yams. Of these, *Yam mosaic virus* (YMV, genus *Potyvirus*), *Yam mild mosaic virus* (YMMV, genus *Potyvirus*) and *Dioscorea* bacilliform viruses (DBVs, genus *Badnavirus*) are widespread in West Africa and YMV has been shown to cause important diseases in yam [3,8,9,10]. YMV infection is associated with a range of symptoms, including mosaic, chlorotic leaf discoloration, green vein banding, and leaf deformation, leading to reduced tuber yield. Infections caused by yam badnaviruses have been linked to symptoms of leaf distortions and veinal chlorosis, although the majority of infected plants show no marked symptoms [6,11].

The use of virus-free (“clean”) planting material is the only efficient method of controlling these virus diseases. However, production and distribution of clean seed yams is hampered by the absence of a formal seed yam certification system [2,12] and the limited knowledge of the diversity of viruses infecting yam. With the increased use of high-throughput sequencing (HTS) technologies and associated bioinformatic pipelines, new virus species and isolates infecting yam are increasingly being discovered [7,13,14,15]. Although the biological impact of these new viruses and isolates is still unknown, their discovery poses a threat to sustainable yam production in West Africa and international exchange of promising breeding lines. More studies are needed to better understand the diversity of viruses in yam that will contribute to the development of efficient and cost-effective diagnostic tools. These will help to make rapid decisions on the health status of yam planting material.

In the present study, we describe and characterize the complete genome sequence of a new virus species present in yam. The new virus, tentatively named “yam virus Y” (YVY), represents a putative new species of the family *Betaflexiviridae*, extending our knowledge on the diversity of viruses present in yam. Our study shows that YVY could be detected in several West African yam plants also infected with YMV and displaying symptoms associated with mosaic disease. Further work is needed to understand the biology of YVY, its association with any symptoms and importance on yield, and implications for seed production.

## 2. Results

### 2.1. High Throughput Sequencing (HTS) Analysis

The HTS of *D. rotundata* cv. Danicha sample yielded 48,934,725 reads. After quality trimming and removing reads corresponding to the yam host, RNA-seq datasets were de novo assembled for genome reconstruction. A total of 43,710 contigs were obtained. Near-complete and complete genomes of known *Yam mosaic virus* (YMV), Dioscorea mosaic-associated virus (DMaV) RNA1 and RNA2, and yam badnavirus were obtained by directly mapping assembled contigs to a custom-made database containing viral reference sequence genomes publicly available from the NCBI GenBank.

Contigs that did not match to any known viruses were annotated using BLASTn/x. Using BLASTn, one contig (7324 nucleotides (nt) long) showed a nucleotide identity score of 69% (12% sequence coverage), corresponding to the partial viral replicase region of *Nerine latent virus* (NeLV, JQ395043). At the amino acid level and using BLASTx, the predicted protein sequence showed homologies with members of the *Betaflexiviridae* family. Conserved domains of a viral methyltransferase (Met), a helicase (Hel), and a RNA-dependent RNA polymerase (RdRp) were identified with amino acid sequence identity levels of 46% (58% sequence coverage) to *Apple stem pitting virus* (GenBank accession ARQ84116).

**Figure 1:**
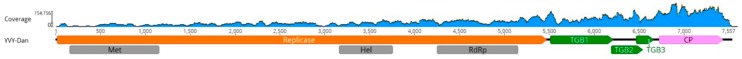
Schematic representation of the genome organization of yam virus Y isolate Danicha (YVY-Dan). Five predicted open reading frames (ORFs) are shown: ORF1 = replicase (orange); ORF2, ORF3, and ORF4 = triple gene blocks (TGB) 1, 2, and 3, respectively (green); ORF 5 = coat protein (CP) (pink). Conserved motifs for viral methyltransferase (Met, pfam 01660), viral helicase_1 (Hel, pfam 01443), RNA-dependent RNA polymerase_2 (RdRp, pfam 00978) are shown in grey. A graphic coverage plot spanning the entire sequence is shown above the YVY-Dan genome.

The draft assembly was then further extended using the Geneious iterative assembler with ten iterations. A single continuous sequence of 7557 nt was obtained representing the complete genome sequence of a new virus, hereafter named “yam virus Y, isolate Danicha” (YVY-Dan; GenBank accession number MK782911) (Figure 1). The raw RNA-seq reads of the Danicha sample were remapped to this new complete virus genome. The new virus genome is supported by 7.7% (3.8 million reads) of total reads and a mean coverage depth of 36.8X.

To confirm that the new viral sequence was not an artefact of the HTS analysis and further prove the presence of YVY-Dan sequence in yam, we performed a long reverse transcription polymerase chain reaction (RT-PCR) using primer “YVY-RdRp1-PF” (Materials and Methods section) and oligo-dT (18 mer) as reverse primer. A fragment of the expected size (~3.5 kb) from the partial RdRp region to the poly-A 3’ end was obtained (Supplementary Materials, Figure S1).

To investigate the possible existence of another YVY isolate, RNA-Seq data from a six weeks old *D. rotundata* cv. Makakusa tissue culture plant showing mild mosaic and chlorotic symptoms obtained by Bömer et al. [7] was analyzed. Over 38 million reads were generated for this Makakusa sample and assembled in to 117,034 contigs using Trinity software. RNA-Seq data revealed the presence of complete genomes of novel badnavirus and YMV isolates [7]. A targeted BLAST search against viral sequences of the *Betafexiviridae* family identified one contig (7584 nt long) displaying conserved domains of a viral Met, Hel, RdRp and Flexi-CP and with amino acid sequence identity levels of 46% (56% sequence coverage) to *Apple stem pitting virus* (GenBank accession ARQ84116). This sequence represents another new complete genome of YVY referred to “yam virus Y isolate Makakusa” (YVY-Mak; GenBank accession number MK782910). YVY-Mak represents 6.6% (2.3 million reads) of the total RNA-seq reads and is covered at a depth of 42X.

### 2.2. Genome Organization

The genomes of YVY-Dan and YVY-Mak are 7557 nt and 7584 nt long, respectively, excluding the 3’ polyA tail. Genomes encode for five putative ORFs, including a triple gene block (TGB) (Table 1) characteristic of members of the family *Betaflexiviridae*. ORF1 encodes a putative viral replicase protein with an estimated mass of 208 kDa. Three conserved domains typical for *Betaflexiviridae* [16] were identified within the ORF1 protein: a viral methyltransferase domain (Met, pfam 01660); a viral helicase 1 domain (Hel, pfam 01443); and an RNA-dependent RNA polymerase 2 domain (RdRp, pfam 00978) (Figure 1). ORF2, ORF3, and ORF4 constitute the triple gene block (TGB) and encode the putative proteins TGB1 (26 kDa), TGB2 (13 kDa), and TGB3 (7 kDa), respectively. These three proteins are believed to be involved in viral cell-to-cell movement [16]. ORF5 encodes for a putative coat protein (CP, 27 kDa) and contains a conserved motif (Flexi-CP, pfam 00286 domain) that is typically conserved in all filamentous viruses [17].

According to the International Committee on Taxonomy of Viruses (ICTV), the demarcation criteria for isolates of different species in the family *Betaflexiviridae* is less than 72% nucleotide (nt) or 80% amino acid (aa) identity in either the complete CP (ORF5) or replicase (ORF1) genes [18]. For the replicase gene, YVY-Dan and YVY-Mak showed the highest sequence identity to *Apple stem pitting virus* (ARQ84116) with 46% aa identity (79% sequence coverage). For the CP gene, both isolates shared 33% aa sequence identity (88% sequence coverage) to *Cherry twisted leaf-associated virus* (AHA59520). The amino acid sequence identity level of both YVY-Dan and YVY-Mak is well below the demarcation criteria. Similar results are obtained with the nucleotide sequences of both replicase and CP genes. Based on these results and when comparing the nucleotide and amino acid sequences of different genes between the two isolates (Table 1), YVY should be considered as a novel species of the family *Betaflexiviridae*.

### 2.3. Phylogenetic Analysis

**Figure 2:**
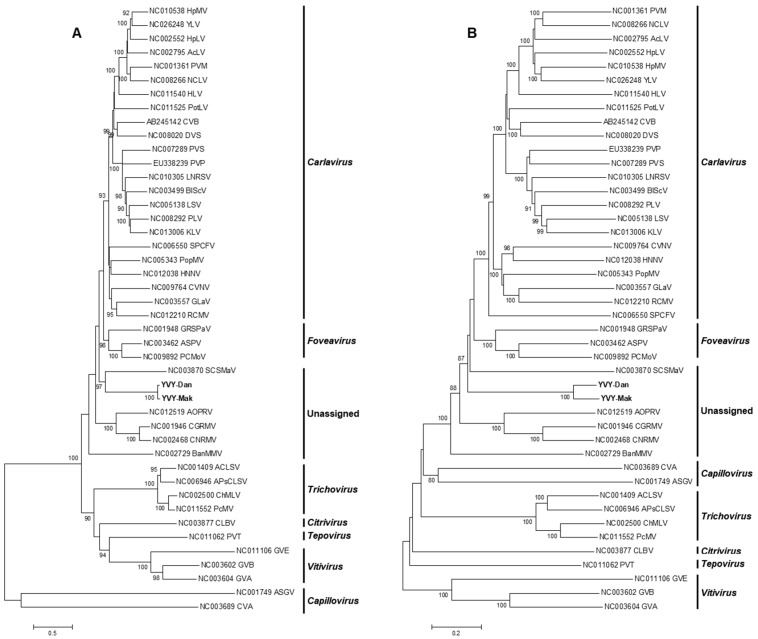
Phylogenetic trees based on (**A**) the amino acid sequences of the entire replicase protein and (**B**) full-genome sequences of YVY-Dan and YVY-Mak and members of the family *Betaflexiviridae*. Bootstrap analysis was performed with 1000 replicates and the cut-off value was 85%. The scale bar represents the number of amino acid and nucleotide substitutions per site. YVY-Dan and YVY-Mak are indicated in bold.

Phylogenetic trees were generated based on the deduced amino acid sequences of the entire replicase protein (Figure 2a) of YVY-Dan and YVY-Mak and of members of the family *Betaflexiviridae*. Phylogenetic analysis indicated that isolates obtained in this study (YVY-Dan and YVY-Mak) form a well-supported clade and group together with members of unassigned species in the family *Betaflexiviridae*, the closest relative being *Sugarcane striate mosaic-associated virus* (SCSMaV). YVY-Dan and YVY-Mak share the highest replicase amino acid and nucleotide identity (30% and 47%, respectively) with SCSMaV. Phylogenetic analysis based on the full-genome sequences (Figure 2b) resulted in a similar topology. To check for possible recombination events, the full genomes of YVY-Dan and YVY-Mak were aligned with other full genomes of members of the family *Betaflexiviriade*. No recombination events were detected for the two YVY isolates (data not shown).

### 2.4. Prevalence and Diversity of the New Virus

Based on the complete genome sequences of YVY, new specific PCR primers targeting a 790 bp region of the RdRP gene and a 788 bp region comprising the CP-3′UTR were designed (Materials and Methods section). These primers were used to screen the presence of the new virus by RT-PCR in 55 yam breeding lines and landraces actively growing in screenhouses at the Natural Resources Institute (NRI, Greenwich, UK), the International Institute of Tropical Agriculture (IITA, Ibadan, Nigeria) and the Council for Scientific and Industrial Research -Crops Research Institute (CSIR-CRI, Accra, Ghana) (Supplementary Materials, Table S1). Results of the RT-PCR showed that 31 samples tested positive for YVY (Table 2) using the primers targeting the CP-3′UTR region, whilst only 27 of these scored positive with the RdRp primer set. The yam samples were also tested for the presence of YMV by RT-PCR using the primers designed by Mumford and Seal [19]. Interestingly, 31 samples were positive for YMV (Table 2). Among these positive samples, 23 showed a mixed infection with YVY. Plants singly infected with YVY were generally asymptomatic, although one symptomatic but YMV-negative sample was found positive for YVY. Plants infected with YMV alone or YMV and YVY in combination were symptomatic.

Eight RT-PCR products from the CP-3′UTR region were sequenced and deposited in GenBank (MK895044-MK895051). A multiple sequence alignment was performed with these sequences using ClustalW in Geneious software. Before further analysis, the CP-3′UTR region was trimmed to the CP coding region (711 nt), not including primer sequences. Nucleotide identity sequencing analysis of the CP gene showed that the different isolates share identities between 84% and 97% with YVY-Dan and 83% to 85% with YVY-Mak (Supplementary Materials, Table S2).

**Figure 3:**
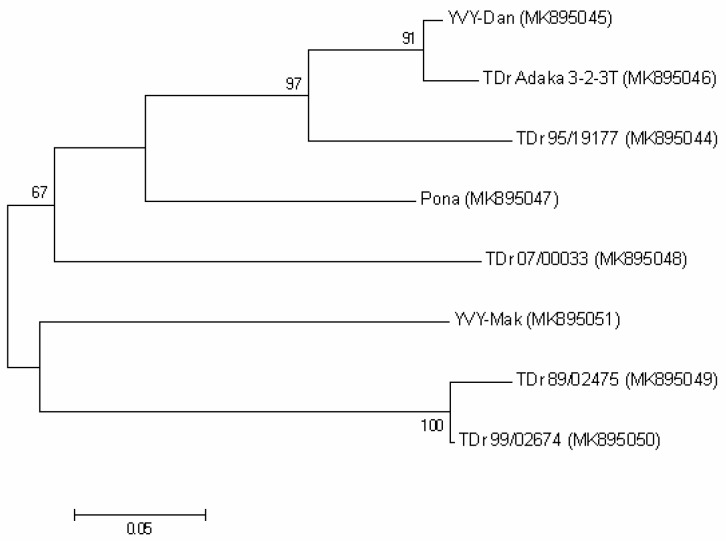
Maximum likelihood phylogenetic tree based on the nucleotide sequences of the CP gene of YVY isolates. GenBank accession numbers are in brackets. These sequences were obtained from yam accessions growing in a quarantine aphid-proof glasshouse at NRI, United Kingdom (YVY-Dan; TDr 95/19177; Pona; TDr 07/00033; YVY-Mak; TDr 89/02475; TDr 99/02674) and IITA, Nigeria (TDr Adaka 3-2-3T). Bootstrap analysis was performed with 1000 replicates and the cut-off value was 65%. The scale bar represents the number of nucleotide substitutions per site.

Phylogenetic analysis based on the CP gene (Figure 3) indicates that YVY isolates cluster in at least 2 different groups; 1 clade represented by YVY-Dan and the other by YVY-Mak. No evidence of recombination was found in this alignment.

## 3. Discussion

In this study, we identified a novel virus, tentatively named “yam virus Y” (YVY), in *D. rotundata* yam samples. The complete genome sequences of two isolates, YVY-Dan and YVY-Mak, were obtained by HTS analysis. The genome of YVY contains five ORFs. ORF1 encodes large replication-associated protein, ORFs2, 3, and 4 constitute the putative triple gene block proteins associated with viral cell-to-cell movement in plants [16] and ORF5 encodes a putative coat protein. The HTS analysis showed a coverage depth biased towards the 3′-end of the genome sequences, which might have been caused by the library preparation protocol [7,20]. Nevertheless, the average coverage depth of both YVY isolates obtained in our analysis is similar to the ones reported by others [21,22].

Sequencing and phylogenetic analysis showed that the virus sharing the highest pairwise identity to both YVY isolates was SCSMaV, an unassigned species of the family *Betaflexiviridae*. The degree of similarity (30% nt and 47% aa on the replicase gene) is well below the demarcation criteria thresholds recommended for all members of the family *Betaflexiviridae* (less than 72% nt or 80% aa identity in either the complete CP or replicase genes), suggesting that YVY should be considered as a new virus species in the family *Betaflexiviridae*. Phylogenetic analysis based on the CP gene indicate that YVY isolates are grouped in at least two different groups. More isolates are needed to better understand the importance of the diversity of YVY isolates.

Yams are a major staple and play a critical role in food security and income generation for small holder farmers in West Africa. Nigeria and Ghana are the major producers and exporters of yam, respectively, and as a consequence several efforts have been made to boost yam production in these countries. The Ghanaian government recently introduced a policy that is geared towards increasing food productivity and ensuring food security for the country, which resulted in a higher demand for high-quality seed yam. Also, the “Yam Improvement for Income and Food Security in West Africa” (YIIFSWA) project [23] will develop a formal seed yam seed system in Nigeria and Ghana to deliver high quality and certified seed yam tubers to small holder farmers. The discovery of YVY might have implications for these national and international programs supporting the multiplication and delivery of virus-free certified planting material to small holder farmers.

The limited survey of viruses in yam collections in the United Kingdom, Nigeria and Ghana showed that YVY is widespread in yam. The new virus was generally found in symptomatic plants that were YMV positive (except in one sample, TDr Kukrupa, which showed symptoms of mosaic disease but tested negative for YMV; Table S1). YVY, however, was also detected in asymptomatic and YMV negative plants. Our results indicate that there is a strong correlation (71%) between YVY and YMV detection. Based on the few samples analysed in our study, it is still unclear what the role of YVY might be in disease development, but it is certain that YVY does pose a challenge for the international exchange of yam breeding material.

Further studies are required to understand the biology, epidemiology and economic significance of YVY. Such studies should address the ability of YVY to cause symptoms, its effects on yield and any synergistic interaction with commonly co-infecting viruses, such as YMV, YMMV and badnaviruses. Our study provides the initial knowledge required and a first set of diagnostic tools (i.e., RT-PCR primers) to enable us to investigate these widespread viruses further in the future.

## 4. Materials and Methods

### 4.1. Plant Material

Yams (*D. rotundata*) used in this study were obtained from the Natural Resources Institute (NRI, United Kingdom), the International Institute of Tropical Agriculture (IITA, Nigeria) and the Council for Scientific and Industrial Research—Crops Research Institute (CSIR-CRI, Ghana) collections.

**Figure 4:**
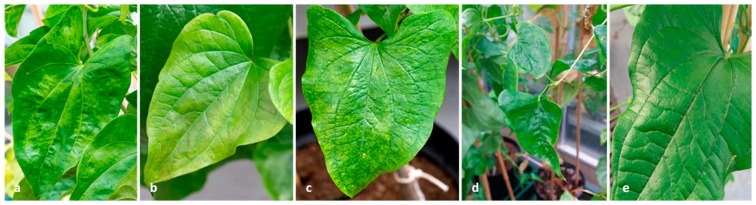
Yam leaf samples used in this study showing typical yam mosaic disease symptoms; (**a**) *D. rotundata* TDr 99/02674 showing mosaic symptoms; (**b**) *D. rotundata* cv. Adaka showing chlorotic leaf discoloration and (**c**) mottling; (**d**) *D. rotundata* TDr 00/00168 showing leaf deformation; (**e**) Asymptomatic *D. rotundata* cv. Adaka. (**a**–**d**) Plants tested positive for YMV, whereas YVY was detected in all the plants (**a**–**e**).

The NRI samples consisted of YMV-infected and uninfected (Figure 4) breeding lines and landraces of *D. rotundata* (*n* = 15) actively growing in a quarantine aphid-proof glasshouse as described by Mumford and Seal [19] and from tissue culture [7]. The YMV infection status of these samples was known and reported before [10,24,25]. *D. rotundata* landrace cv. Danicha showing viral symptoms was used for HTS analysis.

Yam breeding lines and landraces of *D. rotundata* from IITA (*n* = 25) and CSIR-CRI (*n* = 15) were collected and analyzed for the presence of YVY.

### 4.2. RNA Extraction and High Throughput Sequencing (HTS) 

For HTS analysis, total RNA was extracted from leaf tissues of *D. rotundata* cv. Danicha using a modified cetyltrimethyl ammonium bromide (CTAB) method combined with the RNeasy Plant Mini Kit (Qiagen GmbH, Hilden, Germany) [7]. Total RNA was quantified using a NanoDrop 2000 spectrophotometer (Thermo Scientific, Loughborough, UK) and analyzed using the Agilent 2100 Bioanalyzer (Agilent Technologies, Abingdon, UK) to check their RNA integrity number (RIN). The RNA sample was then sent to the Earlham Institute (Norwich, UK) for cDNA libraries preparation using the Illumina TruSeq RNA library kit and sequencing on an Illumina HiSeq4000 platform.

RNA-seq reads were trimmed to remove low quality sequences using Trimmomatic version 0.36 [26]. Trimmed reads were mapped to the genome sequence of *D. rotundata* [27] using Magic-Blast [28]. Trinity version 2.5.1 [29] was used to de novo assemble unmapped reads into longer contiguous sequences (contigs). The resulting contigs were mapped to a customized National Centre for Biotechnology Information (NCBI) viral reference sequences database (downloaded locally on 4 December 2018) using Magic-Blast and subjected to BLASTn and BLASTx searches against the plant virus database of the NCBI GenBank.

### 4.3. Reverse Transcription Polymerase Chain Reaction (RT-PCR)

For YMV and YVY detection by RT-PCR, total nucleic acids were extracted from 100 mg leaf tissue using a modified CTAB method previously described by Abarshi et al. [30].

The presence of YMV was confirmed by RT-PCR using the primer pair YMV-CP-1F/YMV-UTR-1R [19], which amplify a 586 bp and region comprising the coat protein (CP) gene and the 3′ UTR region of the YMV genome. To confirm YVY infection new primers were designed in this study (Table 3) based on the complete genome sequences obtained from HTS.

The RT-PCR protocol was set up in 20 µL reactions containing 1XDreamTaq buffer (Thermo Scientific, UK), 0.25 mM of each dNTP (Thermo Scientific, UK), 0.2 µM of each primer, 1 U DreamTaq DNA polymerase (Thermo Scientific, UK), 2.5 U AMV Reverse Transcriptase (Promega, UK) and 1 µL of purified RNA as a template. Tubes were then subjected to thermal cycling consisting of a RT step at 50 °C for 10 min, followed by 95 °C for 4.5 min and 35 cycles of 95 °C for 30 s, 55 °C for 1 min and 72 °C for 1 min, and one final cycle of 72 °C for 10 min. RT-PCR amplification products were analyzed by agarose gel electrophoresis, purified using the GeneJET PCR Purification Kit (Fermentas, UK) and Sanger sequenced by the Source BioScience sequencing service (Cambridge, UK).

### 4.4. Phylogenetic and Sequence Analysis

Putative open reading frames (ORFs) were identified using the NCBI ORF finder (https://www.ncbi.nlm.nih.gov/orffinder/). Conserved domains of the putative gene products were searched using the conserved domain tool (http://www.ncbi.nlm.nih.gov/Structure/cdd/wrpsb.cgi). The full-length genome and partial nucleotide and deduced amino acid sequences of YVY were aligned with other viral sequences from *Betaflexiviridae* family obtained from GenBank database. Multiple sequence alignments were performed using Geneious software v10.2.6 (Biomatters Ltd., Auckland, New Zealand). Phylogenetic analysis for full genome, replicase and CP genes were performed in MEGA7 [31] using the best maximum likelihood-fitted model for each sequence alignment. The robustness of each tree was determined by generating a bootstrap consensus tree using 1000 replicates. Virus sequences obtained from GenBank were used for comparative analyses and accession numbers are shown in the phylogenetic trees. Recombination analysis was performed using the RDP4 software package with default settings [32].

## 5. Conclusions

Our study describes the presence of a novel virus of unassigned taxonomic status that is indicated to be widespread in West African yams. YVY was detected in both symptomatic and asymptomatic plants, but symptomatic plants were always positive to YMV, an endemic virus known to be responsible for mosaic disease in West Africa. Yams infected by YVY alone were generally asymptomatic. This data will be seminal for further studies regarding the biological and epidemiological features of this novel virus. It will be paramount to strengthen the yam seed certification system in order to prevent the spread of viral diseases that might threaten food and income security to small holder farmers in West Africa.

## Figures and Tables

**Table 1:** Nucleotide and amino acid length of putative genes and identity percentages (%) between the YVY-Dan and YVY-Mak isolates described in this study.

ORFs ^1^	Gene ^2^	Length (nt/aa)		% Identity (nt/aa) YVY-Dan vs. YVY-Mak
		YVY-Dan	YVY-Mak	
1	Replicase	5451/1816	5454/1817	83/93
2	TGB1	702/233	702/233	84/91
3	TGB2	348/115	348/115	86/92
4	TGB3	198/65	198/65	86/89
5	CP	711/236	711/236	85/94

^1^ ORF: open reading frames. ^2^ Replicase: viral replicase; TGB1: triple gene block 1; TGB2: triple gene block 2; TGB3: triple gene block 3; CP: coat protein.

**Table 2:** Number of yam plants infected by YVY and YMV.

Virus	YMV +	YMV −	Total
YVY +	23 (42%)	8 (15%)	31
YVY −	8 (15%)	16 (29%)	24
Total	31	24	55

Note: + and − indicate presence and absence of virus, respectively.

**Table 3:** Primers used for detection of YVY.

Name	Sequence (5′–3′)	Product Size (bp)	Location
YVY-RdRp1-PF	GTAATTGAAAATCACAGTGAGC	790	RdRp
YVY-RdRp1-PR	CTTCAAGTGCATAATTGTCTAT		
YVY-CP-F	TTGATTAGTTAAGTATTTAGC	788	CP-3′UTR
YVY-CP-R	CCAGTTTTTCCTGCTGGCAAAC		

RdRp: RNA-dependent RNA polymerase; CP: coat protein; UTR: untranslated region.

## Supplementary Materials

The following are available online at https://www.mdpi.com/2223-7747/8/6/167/s1. Table S1: Results of screening for the presence of YVY and YMV on different yam accessions by RT-PCR; Table S2: Nucleotide identity matrix of YVY isolates based on the CP gene; Figure S1: Resolution of the long RT-PCR amplification products obtained with primer “YVY-RdRp1-PF” (Table 3) and oligo-dT (18 mer) as reverse primer. 1: *Dioscorea rotundata* (TDr) Danicha; 2: TDr Aloshi; MW: GeneRuler™ 1 kb Plus DNA Ladder (Thermo Scientific, UK); NTC: non-template (water) control.
