# Supplementary material for: Molecular Characterization of a New Virus Species Identified in Yam (Dioscorea spp.) by High-Throughput Sequencing
Source: Plants (Basel). 2019 Jun 11;8(6):167. doi: 10.3390/plants8060167 (PMC6630666; doi:10.3390/plants8060167)
Supplement: Supplementary file 1 [file plants-08-00167-s001.zip › Supplementary Materials Table S1_Final.docx]

**Table S1**. Results of screening for the presence of YVY and YMV on different yam accessions by RT-PCR.

| **Yam accession^1^** | **Source** | **Status^2^** | **YMV** | **YVY** |
| --- | --- | --- | --- | --- |
| TDr Adaka | NRI, UK | Symptomatic | +^3^ | + |
| TDr Aloshi | NRI, UK | Asymptomatic | -^3^ | - |
| TDr Ame | NRI, UK | Symptomatic | + | - |
| TDr Ogini | NRI, UK | Asymptomatic | - | - |
| TDr Ogoja | NRI, UK | Asymptomatic | - | - |
| TDr Pona | NRI, UK | Symptomatic | + | - |
| TDr Adaka 1-2-1U^4^ | IITA, Nigeria | Symptomatic | + | + |
| TDr Adaka 2-2-2U^4^ | IITA, Nigeria | Symptomatic | + | + |
| TDr Adaka 3-2-1T^4^ | IITA, Nigeria | Asymptomatic | - | - |
| TDr Adaka 3-2-3T^4^ | IITA, Nigeria | Symptomatic | + | + |
| TDr Adaka 3-2-2U^4^ | IITA, Nigeria | Symptomatic | + | + |
| TDr Adaka 2-2-3Y^4^ | IITA, Nigeria | Asymptomatic | - | + |
| TDr Aloshi-22 | IITA, Nigeria | Asymptomatic | - | - |
| TDr Aloshi-14 | IITA, Nigeria | Asymptomatic | - | - |
| TDr Kpamyo | IITA, Nigeria | Asymptomatic | - | - |
| TDr Asiedu | IITA, Nigeria | Asymptomatic | - | - |
| TDr Swaswa | IITA, Nigeria | Asymptomatic | - | - |
| TDr Pona 13 | CSIR-CRI, Ghana | Asymptomatic | - | - |
| TDr Mankrong Pona 94 | CSIR-CRI, Ghana | Symptomatic | + | - |
| TDr Mankrong Pona 94 | CSIR-CRI, Ghana | Asymptomatic | - | - |
| TDr CRI Pona | CSIR-CRI, Ghana | Symptomatic | + | + |
| TDr Pona S13 | CSIR-CRI, Ghana | Symptomatic | + | + |
| TDr Labariko | CSIR-CRI, Ghana | Symptomatic | + | - |
| TDr Kperino Dente | CSIR-CRI, Ghana | Symptomatic | + | - |
| TDr Kukrupa | CSIR-CRI, Ghana | Symptomatic | - | + |
| TDr Labreko | CSIR-CRI, Ghana | Symptomatic | + | - |
| TDr CRI-Pona | CSIR-CRI, Ghana | Asymptomatic | - | + |
| TDr CRI-Pona | CSIR-CRI, Ghana | Symptomatic | + | + |
| TDr CRI-Pona (M 2.1) | CSIR-CRI, Ghana | Symptomatic | + | + |
| TDr Kpuno | CSIR-CRI, Ghana | Symptomatic | + | + |
| TDr Pona (S23) | CSIR-CRI, Ghana | Symptomatic | + | + |
| TDr 00/00168 | NRI, UK | Symptomatic | + | + |
| TDr 02/00515 | NRI, UK | Symptomatic | + | + |
| TDr 03/00196 | NRI, UK | Symptomatic | + | + |
| TDr 07/00033 | NRI, UK | Symptomatic | + | + |
| TDr 89/02475 | NRI, UK | Symptomatic | + | + |
| TDr 89/02665 | NRI, UK | Asymptomatic | - | - |
| TDr 95/19177 | NRI, UK | Symptomatic | + | + |
| TDr 96/00604 | NRI, UK | Symptomatic | + | + |
| TDr 99/02674 | NRI, UK | Symptomatic | + | + |
| TDr 4914 | IITA, Nigeria | Symptomatic | + | - |
| TDr 4984 | IITA, Nigeria | Symptomatic | + | + |
| TDr 5008 | IITA, Nigeria | Symptomatic | + | + |
| TDr 5011 | IITA, Nigeria | Symptomatic | + | + |
| TDr 5066 | IITA, Nigeria | Symptomatic | + | + |
| TDr 5052 | IITA, Nigeria | Asymptomatic | - | - |
| TDr 5054 | IITA, Nigeria | Asymptomatic | - | + |
| TDr 5058 | IITA, Nigeria | Asymptomatic | - | + |
| TDr 5063 | IITA, Nigeria | Asymptomatic | - | - |
| TDr 5108 | IITA, Nigeria | Asymptomatic | - | + |
| TDr 5008 | IITA, Nigeria | Asymptomatic | - | + |
| TDr 89/02665 | IITA, Nigeria | Symptomatic | + | - |
| TDr 09/00058 | IITA, Nigeria | Asymptomatic | - | - |
| TDr 97/18544 | IITA, Nigeria | Asymptomatic | - | - |
| TDr 95/19177 | CSIR-CRI, Ghana | Asymptomatic | - | + |

^1^ TDr: *Dioscorea rotundata* accession

^2^ Symptom status: Symptomatic plants showed symptoms related to mosaic disease (mosaic, chlorotic leaf discoloration, green vein banding, leaf deformation); Asymptomatic plants showed no symptoms

^3^ +: Positive result; −: negative result

4 Tissue culture plants
