# Supplementary figures and images for: Molecular Characterization of a New Virus Species Identified in Yam (Dioscorea spp.) by High-Throughput Sequencing
Source: Plants (Basel). 2019 Jun 11;8(6):167. doi: 10.3390/plants8060167 (PMC6630666; doi:10.3390/plants8060167)

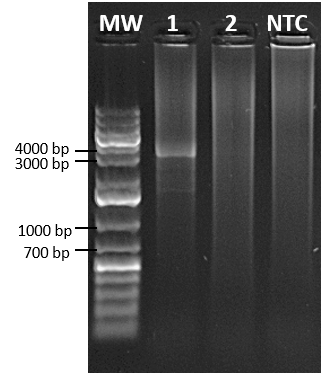

Supplement: Supplementary file 1 [file plants-08-00167-s001.zip › Figure S1 - Long RT-PCR.tif]
